# Incidence of Breast Cancer in Fars Province, Southern Iran: A Hospital-Based Study

**Published:** 2012-01

**Authors:** Davood Mehrabani, Amir Almasi, Mahin Farahmand, Z Ahrari, Abbas Rezaianzadeh, Golnoush Mehrabani, Abdol Rasoul Talei

**Affiliations:** 1 Stem Cell and Transgenic Technology Research Center, Shiraz University of Medical Sciences, Shiraz, Iran.; 2 Department of Epidemiology, School of Public Health, Arak University of Medical Sciences, Arak, Iran.; 3 Office of Vice Chancellor for Health Affairs, Shiraz University of Medical Sciences, Shiraz, Iran; 4 Department of Epidemiology, School of Public Health, Shiraz University of Medical Sciences, Shiraz, Iran; 5 Gastroenterohepatology Research Center, Shiraz University of Medical Sciences, Shiraz, Iran; 6 Department of Surgery, School of Medicine, Shiraz University of Medical Sciences, Shiraz, Iran.

**Keywords:** Incidence, Breast cancer, Iran

## Abstract

**BACKGROUND:**

Breast cancer is still considered as one of the most common female cancers worldwide regardless of the countries’ level of development. This study determines the incidence of breast cancer in Fars Province, Southern Iran.

**METHODS:**

This study used patients' records from Shiraz University of Medical Sciences Cancer Registry Centre, which is a Hospital-Based Registry of Nemazee Hospital. Data were recorded based on International Classification *of* Diseases for Oncology (ICD-O) and compromised all invasive cancers in ICD-10 categories of C-00 to C-80. The findings were shown as the number of cases by site (ICD-10) and gender, with crude incidence (CRs), age-specific incidence and age-standardized incidence rates (ASRs) per 100,000 persons per year, performed by direct method using the world standard population.

**RESULTS:**

The age group of 40-49 years had the highest rate of breast cancer and naturally most cases were post-menopause ones. Most cases were diagnosed in moderate differentiated state with an increasing trend. Early diagnosis of in situ neoplasms has not increased over time in comprised with malignant cases. The number of diagnosed cases has sharply increased after year 2004 especially during post-menopause period.

**CONCLUSION:**

As the number of diagnosed cases has increased during post-menopausal period, screening and health programs seem necessary for menopause women.

## INTRODUCTION

Breast cancer is still considered as one of the most common female cancers worldwide regardless of the countries’ level of development.^1^ Its prevalence in Europe and USA was reported 8-10%, however, the lowest prevalence was noticed in Asian countries as 1%.^2^ In Iran, the prevalence of breast cancer was reported as 6.7/1000 in 2002 and in ranking is the first among female malignancies^3^^,^^4^ comprising 24.4% of all neoplasms^5^ with a crude incidence rate of 17.81^3^ and an ASR of 23.65^3^ in the year of 2006. In Fars Province, southern Iran, it is the first common cancer in females with a crude incidence of 11.58 and ASR of 18.06^6^ with a 5 years survival rate of 58%.^7^^,^^8^

However, several studies specifically described the clinico-pathologic features, stages, and age distributions of breast cancer.^3^^-^^5^^,^^9^^-^^17^ It is difficult to predict the present and future patterns of breast cancer in Iran and carry out the most appropriate preventive and therapeutic measures to decrease the burden of the disease.^18^ In addition, concerning the high mortality rate of breast cancer in developing countries^15^ and its increasing incidence, an epidemiologic evaluation analysis focusing on the recent shift in the age of presentation should be considered for primary and secondary prevention of this cancer.^17^^,^^19^

Iran has a total population of just over 75 million and almost all studies of breast cancer in Iran are from the capital, Tehran with a population of approximately 14 million. Southern Iran has a population of approximately 4 million.^6^ Geographical variations in incidence and mortality rates of breast cancer suggest that the known risk factors for breast cancer may vary in different parts of the world and that environmental factors are of greater importance than genetic factors.^20^ For instance, in Iran; it has been shown that even after adjusting for age, young women are at relatively higher risk for developing breast cancer than are their Western counterparts.^9^

Breast cancer begins in breast tissue, which is made up of glands for milk production, called lobules, and the ducts that connect lobules to the nipple. The remainder of the breast is made up of fatty, connective, and lymphatic tissue.^21^ Breast cancer incidence and death rates generally increase with age. 

During 2002- 2006, 95% of new cases and 97% of breast cancer deaths occurred in women aged 40 and older.^21^ Many of the known breast cancer risk factors, such as age, family history, age at first full-term pregnancy, early menarche, late menopause, and breast density, are not easily modifiable. However, other factors associated with increased breast cancer risk (postmenopausal obesity, use of combined estrogen and progestin menopausal hormones, alcohol consumption, and physical inactivity) are modifiable. Besides being female, age is the most important risk factor for breast cancer.^21^

In this study we are trying to verify incidence rate of breast cancer in south Iran based on reports from Cancer Registry Center of Nemazee Hospital in Fars Province, Southern Iran and see if reported risk factors of breast cancer have changed. Women aging 15 years and older have been included in this study and several factors associated with breast cancer, like menopause status, pathology information and age, has been taken into consideration. 

## MATERIALS AND METHODS

This study used patients' records from Shiraz University of Medical Sciences Cancer Registry Centre, which is a Hospital-Based Registry of Nemazee Hospital, a tertiary care centre which delivers oncology services to a population of approximately four millions. In an active system, data were recorded in a sheet and coded based on ICD-O and all duplicate reports were eliminated. The personnel interviewed the patients to receive all information face-to-face. The registered cases comprised all invasive cancers in ICD-10 categories of C-00 to C-80. The cancer registry team actively collected and compiled the data for a period of 6 years from 2001 to 2006 from 4 hospitals of Shiraz University of Medical Sciences including Nemezee, Faghihi, Chamran and Zeynabieh hospitals comprising 87.8%, 8.3%, 1.7% and 2.2% of patient-referrals, respectively. As Nemezee Hospital is equipped with radio and chemotherapy centers, it supports most of the referrals. The findings were shown as the number of cases by site (ICD-10) and gender, with crude incidence (CRs), age-specific incidence and age-standardized incidence rates (ASRs) per 100,000 persons per year, performed by direct method using the world standard population. The data were statistically analyzed using SPSS (version 11.5, Chicago, IL, USA), and MS EXCEL (Microsoft, Raymond, WA, USA) softwares. A P value less than 0.05 was considered as significant. The primary site and morphology data were coded using the ICD-O.45 Information on other variables was coded as advised by International Agency For Research on Cancer (IARC). Women menopause age was considered 47 years based on previous studies in the target population.

## RESULTS

Statistical data about age group of patients is being shown in Table 1. As is obvious from the table, the age group of 40-49 years had the highest rate of breast cancer and naturally most cases were post-menopause ones.

**Table 1:** Statistical data on age group of patients with breast cancer

**Variables**		**Total**	**2001**	**2002**	**2003**	**2004**	**2005**	**2006**
Age	Mean±SD	48.9±12.4	49.03±12.3	48.3±12.1	47±12.7	49.4±12	49.1±12	49.6±13
	Median (min, max)	48 (15-95)	48 (22-95)	48 (22-84)	46 (18-90)	48 (20-84)	48 (19-94)	48 (15-90)
Age groups	15-19	8 (0.4)	0	0	3 (1.1)	0	1 (0.2)	4 (0.8)
	20-29	69 (3.5)	8 (3.5)	2 (2.5)	15 (5.7)	10 (3.5)	16 (3.4)	14 (2.8)
	30-39	359 (18.1)	39 (17.1)	53 (22.3)	48 (18.3)	48 (16.7)	79 (16.6)	92 (18.6)
	40-49	667 (33.6)	83 (36.4)	74 (31.1)	98 (37.3)	99 (34.4)	169 (35.4)	144 (29.1)
	50-59	476 (23.9)	54 (23.7)	56 (23.5)	57 (21.7)	70 (24.3)	125 (26.2)	114 (23.1)
	60-69	243 (12.2)	25 (11)	35 (14.7)	25 (9.5)	40 (13.9)	53 (11.1)	65 (13.2)
	+ 70	166 (8.4)	19 (8.3)	14 (5.9)	17 (6.5)	21 (7.3)	34 (7.1)	61 (12.3)
Pre-menopause		962 (48.4)	107 (46.9)	115 (48.3)	146 (55.5)	138 (47.9)	232 (48.6)	224 (45.3)
Post-menopause		1026 (51.6)	121 (53.1)	123 (51.7)	117 (44.5)	150 (52.1)	245 (51.4)	270 (54.7)
Number of breast cancer cases		1988	228	238	263	288	477	494

As is shown in Table 2, most cases from 2001 to 2006 were diagnosed in moderate differentiated state with an increasing trend. Unfortunately, early diagnosis (in situ neoplasm) has not increased over time in comparison with invasive malignant cases.

**Table 2:** Frequency distribution (%) of breast cancer in Fars Province, during 2001-2006.

**Variables**		**Total**	**2001**	**2002**	**2003**	**2004**	**2005**	**2006**
Grading	Well differentiated	186 (9.4)	10 (4.4)	8 (3.4)	38 (14.4)	36 (12.5)	44 (9.2)	50 (10.1)
	Moderate differentiated	593 (29.8)	24 (10.5)	37 (15.5)	92 (35)	110 (38.2)	154 (32.3)	176 (35.6)
	Poorly differentiated	228 (11.5)	9 (3.9)	V4 (1.7)	42 (16)	32 (11.1)	80 (16.8)	61 (12.3)
	Not determined or applicable	981 (49.3)	185 (81.1)	189 (79.4)	91 (34.6)	110 (38.2)	199 (41.7)	207 (41.9)
Behavior	In situ neoplasm	51 (2.6)	1 (0.4)	2 (0.8)	5 (1.9)	12 (4.2)	19 (4)	12 (2.4)
	Malignant- primary site	1937 (97.4)	227 (9.6)	236 (99.2)	258 (98.1)	276 (95.8)	458 (96)	482 (97.6)
Topography	Nipple	12 (0.6)	1 (0.4)	1 (0.4)	1 (0.4)	4 (1.4)	1 (0.2)	4 (0.8)
	Central portion	64 (3.2)	0	3 (1.3)	15 (5.7)	14 (4.9)	24 (5)	8 (1.6)
	Upper inner qua.	43 (2.2)	2 (0.9)	3 (1.3)	6 (2.3)	4 (1.4)	9 (1.9)	19 (3.8)
	Lower inner qua.	23 (1.2)	0	0	5 (1.9)	4 (1.4)	9 (1.9)	5 (1)
	Upper outer qua.	258 (13)	6 (2.6)	11 (4.6)	51 (19.4)	49 (17)	79 (16.6)	62 (12.6)
	Lower outer qua.	51 (2.6)	2 (0.9)	2 (0.8)	8 (3)	10 (3.5)	17 (3.6)	12 (2.4)
	Axillary tail	2 (0.1)	0	0	0	0	0	2 (0.4)
	Overlapping lesion	47 (2.4)	0	2 (0.8)	6 (2.3)	7 (2.4)	9 (1.9)	23 (4.7)
	Breast , NOS	1488 (74.8)	217 (95.2)	216 (90.8)	171 (65)	196 (68.1)	329 (69)	359 (72.7)
Morphology	Neoplasm, malignant	20 (1)	0	1 (0.4)	0	0	1 (0.2)	18 (3.6)
	Carcinoma, NOS	45 (2.3)	15 (6.6)	6 (2.5)	5 (1.9)	5 (1.7)	11 (2.3)	3 (0.6)
	Mucinous adenocaecinoma	14 (0.7)	2 (0.9)	4 (1.7)	3 (1.1)	0	3 (0.6)	2 (0.4)
	Infiltrating duct carcinoma	1622 (81.6)	180 (78.9)	188 (79)	217 (82.5)	244 (84.7)	381 (79.9)	412 (83.4)
	Medullary carcinoma	118 (5.9)	11 (4.8)	21 (8.8)	15 (5.7)	21 (7.3)	29 (6.1)	21 (4.3)
	Lobular carcinoma	94 (4.7)	17 (7.5)	9 (3.8)	11 (4.2)	12 (4.2)	28 (5.9)	17 (3.4)
	Others	75 (3.8)	3 (1.3)	9 (3.8)	12 (4.6)	6 (2.1)	24 (5)	21 (4.3)
Total		1988	228	238	263	288	477	494

NOS: not otherwise specified

**Fig. 1:**
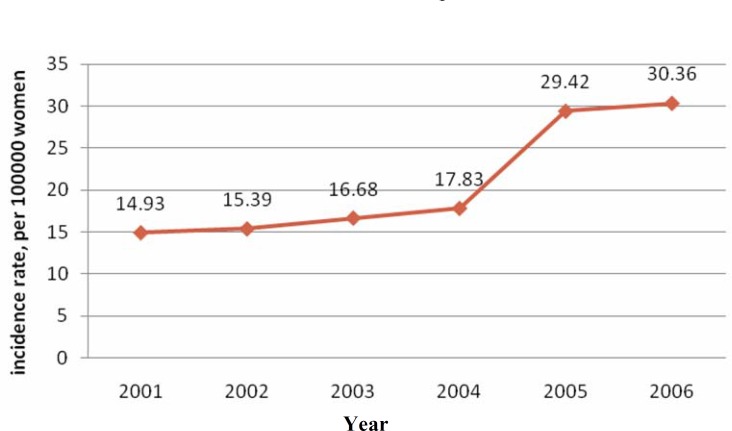
The trend of breast cancer incidence rate from 2001-2006 in Fars provinces.

Figure 1 present the trend of breast cancer in Fars Province during these years demonstrating that the number of diagnosed cases has sharply increased in 2005. 

**Fig. 2:**
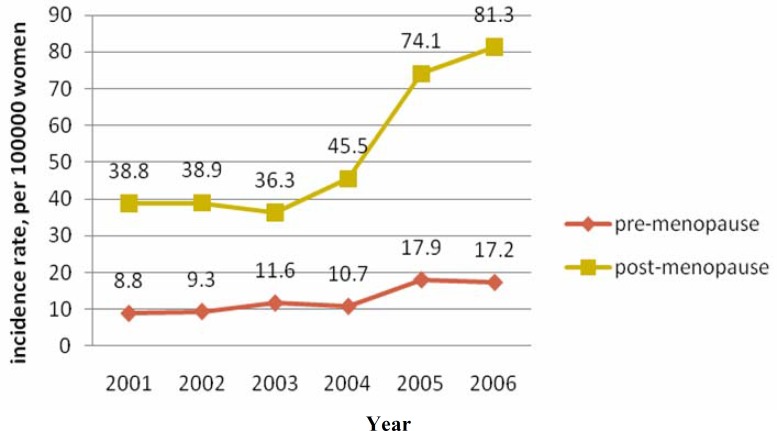
The comparison incidence rate of breast cancer between pre and post menopause women.

Figure 2 clearly describes that number of diagnosed cases has sharply increased after 2004 during post-menopause period. 

## DISCUSSION

Clinico-pathological reports of breast cancer in southern Iran describing the age and stage patterns of patients are so spare^6^^,^^13^ and we could not find any data comparing incidence of the disease in different time periods. In this report we tried to show the incidence of breast cancer in different age group of women and show how some reported risk factors were associated with this cancer, in southern Iran from 2002 to 2006. The incidence of this cancer has sharply increased especially after 2004, mainly after menopausal age.

Some previous reports have shown that age of breast cancer is lower in Iran in comparison with western communities,^9^ and our study confirmed this report. The median age of breast cancer incidence was reported 61 years in United States from 2002 to 2006^22^ but in our study, this age was shown to be 48 years. Our population is a young one and the lower incidence age may be related to this factor. So age standardization was performed to avoid misinterpretations.

The increase was shown in number of cases after menopause age that could be related to the increase in use of estrogen hormones after menopausal age. But these findings should be interpreted with caution as there is no report admitting increase of estrogen hormone use in southern Iran. Although there are some reports showing that use of these hormones can increase risk of breast cancer,^11^^,^^12^ some others reject this finding.^13^^,^^14^ However cause-specific mortality and the increment in incidence in most Asian countries are much higher than in Western countries,^15^ which can be due to increased life expectancy and changes in reproductive and behavioral patterns associated with a heightened breast cancer risk.^16^

As is shown in Table 3, we considered crude and age standardized rate (ASR) of breast cancer in Iran, as 18.9 and 25.06 respectively, but *Musavi et al.*^4^ reported as 17.81 and 23.65. 

**Table 3:** The breast cancer incidence rate in Fars cancer registry compared with other reports.

**Different area **	**Crude**	**ASR of breast cancer**
Fars Cancer Registry (2001-2006)	20.76	19.12
Ardabil Cancer Registry^22^	4.71	7.6
Iran^5^	18.9	25.06
Globocan (2000)^1^	34.9	35.7
South Korea^23^	32.2	26.2
European Union^24^	-	110.3
Switzerland^25^	-	110.5
Europe^26^	-	94.3

As the number of diagnosed cases in our study has increased during post-menopausal period, screening and health programs seem necessary for menopause women in our region.

## References

[1] Parkin DM, Bray F, Ferlay J, Pisani P (2005). Global cancer statistics. Cancer J Clin.

[2] Farooq S, Coleman MP (2005). Breast cancer survival in South Asian women in England and Wales. J Epidemiol Community Health.

[3] Sadjadi A, Nouraie M, Mohagheghi MA, Mousavi-Jarrahi A, Malekezadeh R, Parkin DM (2005). Cancer occurrence in Iran in 2002, an international perspective study. Asian Pac J Cancer Prev.

[4] Mousavi SM, Gouya MM, Ramazani R, Davanlou M, Hajsadeghi N, Seddighi Z (2009). Cancer incidence and mortality in Iran. Ann Oncol.

[5] Goya M (2007). Iranian Annual Cancer Registration Report, 2005/2006.

[6] Mehrabani D, Tabei SZ, Heydari ST, Shamsina SJ, Shokrpour N, Amini M, Masoumi SJ, Julaee H, Farahmand M, Manafi A (2008). Cancer occurrence in Fars Province, Southern Iran. Iran Red Crescent Med J.

[7] Rezaianzadeh A, Peacock J, Reidpath D, Talei A, Hosseini SV, Mehrabani D (2009). Survival analysis of 1148 women diagnosed with breast cancer in Southern Iran. BMC J CAN.

[8] Rajaeefard AR, Baneshi MR, Talei AR, Mehrabani D (2009). Survival models in breast cancer patients. Iran Red Crescent Med J.

[9] Harirchi I, Ebrahimi M, Zamani N, Jarvandi S, Montazeri A (2000). Breast cancer in Iran: A review of 903 case records. Public Health.

[10] Ursin G, Ross RK, Sullivan-Halley J, Hanisch R, Henderson B, Bernstein L (1998). Use of oral contraceptives and risk of breast cancer in young women. Breast Can Res Treat.

[11] Collaborative Group on Hormonal Factors in Breast Cancer (1997). Breast cancer and hormone replacement therapy: Collaborative reanalysis of data from 51 epidemiological studies of 52,705 women with breast cancer and 108,411 women without breast cancer. Lancet.

[12] Heiss G, Wallace R, Anderson GL, Aragaki A, Beresford SA, Brzyski R, Chlebowski RT, Gass M, LaCroix A, Manson JE, Prentice RL, Rossouw J, Stefanick ML (2008). WHI Investigators: Health risks and benefits 3 years after stopping randomized treatment with estrogen and progestin. JAMA.

[13] Mahuri K, Dehghani Zahedani M, Zare S (2007). Breast cancer risk factors in south of Islamic Republic of Iran: A case control study. Eastern Mediter Health J.

[14] Wrensch M, Chew T, Farren G, Barlow J, Belli F, Clarke C, Erdmann CA, Lee M, Moghadassi M, Peskin-Mentzer R, Quesenberry CP Jr, Souders-Mason V, Spence L, Suzuki M, Gould M (2003). Risk factors for breast cancer in population with a high incidence risk. Breast Cancer Res.

[15] Shibuya K, Mathers CD, Boschi-Pinto C, Lopez AD, Murray CJ (2002). Global and regional estimates of cancer mortality and incidence by site: II. Results for the global burden of disease 2000. BMC Cancer.

[16] WHO World Cancer Report 2008. http://www.iarc.fr/en/publications/pdfsonline/wcr/2008/index.php.

[17] Harirchi I, Karbakhsh M, Montazeri A, Ebrahimi M, Jarvandi S, Zamani N, Momtahen AJ, Kashefi A, Zafarghandi MR (2010). Decreasing trend of tumor size and downstaging in breast cancer in Iran: Results of a 15-year study. Eur J Cancer Prev.

[18] Harirchi I, Karbakhsh M, Kashefi A, Momtahen AJ (2004). Breast cancer in Iran: Results of a multicenter study. Asian Pac J Cancer Prev.

[19] Mousavi SM, Harirchi I, Ebrahimi M, Mohagheghi MA, Montazeri A, Jarrahi AM, Gouya MM, Miller AB (2008). Screening for breast cancer in Iran: A challenge for health policy makers. Breast J.

[20] McPherson K, Steel CM, Dixon JM (2000). Breast cancer-epidemiology, risk factors, and genetics. BMJ.

[21] (2009). Breast cancer facts and figures.

[22] Sadjadi A, Malekzadeh R, Derakhshan MH, Sepehr A, Nouraie M, Sotoudeh M, Yazdanbod A, Shokoohi B, Mashayekhi A, Arshi S, Majidpour A, Babaei M, Mosavi A, Mohagheghi MA, Alimohammadian M (2003). Cancer occurrence in Ardabil: results of a population-based cancer registry from Iran. Int J Cancer.

[23] Jung KW, Park S, Kong HJ, Won YJ, Boo YK, Shin HR, Park EC, Lee JS (2010). Cancer statistics in Korea: incidence, mortality and survival in 2006-2007. J Korean Med Sci.

[24] Levi F, Lucchini F, Negri E, La Vecchia C (2007). Continuing declines in cancer mortality in the European Union. Ann Oncol.

[25] Ess S, Savidan A, Frick H, Rageth Ch, Vlastos G, Lütolf U, Thürlimann B (2010). Geographic variation in breast cancer care in Switzerland. Cancer Epidemiol.

[26] Bosetti C, Bertuccio P, Levi F, Chatenoud L, Negri E, La Vecchia C (2011). The decline in breast cancer mortality in Europe: An update (to 2009). Breast.

